# Physical activity and odds of coronary heart disease among Lebanese women

**DOI:** 10.1186/s12889-024-18042-7

**Published:** 2024-02-19

**Authors:** Fatima Ghaddar, Rouba K Zeidan, Pascale Salameh, Françoise Maupas-Schwalm

**Affiliations:** 1 Faculty of Public Health II, Lebanese University, Fanar, Lebanon; 2 Research Institute for Medical and Health Sciences, University of Sharjah, Sharjah, UAE; 3 INSPECT-LB, National Institute of Public Health, Clinical Epidemiology and Toxicology, Beirut, Lebanon; 4 CERIPH, Center for Research in Public Health, Faculty of Public Health, Lebanese University, Mount-Lebanon, Lebanon; 5 Department of Research, Faculty of Pharmacy, Lebanese University, Hadath, Lebanon; 6 Department of Primary Care and Population Health, University of Nicosia Medical School, Nicosia, Cyprus; 7 School of Medicine, Lebanese American University, Byblos, Lebanon; 8 Faculty of Medicine, CHU Toulouse Rangueil, Toulouse, France

**Keywords:** Physical activity, Sedentary behavior, Coronary heart disease, Lebanese women, Case–control study

## Abstract

**Background:**

It is known that physical activity (PA) is protective against cardiovascular morbidity and mortality. However, few studies have examined the association between PA, sedentary lifestyle and coronary heart disease (CHD) in women. This case–control study investigates the relationship between PA and sedentary behavior on CHD odds in Lebanese women over forty.

**Methods:**

One thousand five hundred selected Lebanese women (300 cases and 1200 controls) were included between 2018–2019. Cases were hospitalized women newly diagnosed with CHD, whereas the control groups were free of any heart diseases. Data on socio-demographic, lifestyle, cardiovascular factors, PA and sedentary lifestyle were collected. Multivariate logistic regressions, adjusted for covariates, were performed to investigate the association of PA domains and sedentary behavior with CHD.

**Results:**

A sedentary lifestyle combined with low activity levels increased the odds of CHD. Among cases, 46.7% participated in moderate or vigorous PA against almost 60.3% of controls. 36.3% of coronary patients had more than 10 h/day of sedentary time, with a positive correlation with CHD (adjusted OR: 1.533, 95%CI: 1.046–2.247). Conversely, moderate and high levels (respectively 600–3000 and ≥ 3000 metabolic equivalents [MET]-minutes/week) of domestic/garden PA revealed lower CHD odds (OR: 0.566, 95%CI: 0.396–0.808 and 0.193, 0.065–0.578 respectively). The detrimental influence of sedentary lifestyle appeared to be significantly reversed by weekly moderate PA, especially as weekly sedentary time was less (OR: 0.616, 95%CI: 0.427–0.888/ 6 to10h of sedentary time and OR: 0.537, 95% CI: 0.37–0.779/ ≤ 6 h), and except sedentary time exceeding 10 h daily. Two PA patterns revealed lower CHD odds: transport-related and domestic/garden PA, as early as low amount, even after adjustment for possible confounders.

**Conclusion:**

The current study highlights the importance of combating sedentary behaviors and engaging in regular, easily accessible PA to reduce the odds of coronary disease among aging women. Therefore, better information regarding the benefits of physical activities such as transportation-related activities or gardening would be helpful in enhancing the prevention of CHD in aging women.

## Introduction

Physical activity (PA) is recognized by the American Heart Association as an independent protective factor against coronary heart disease (CHD) [1]. In particular, PA can help lower blood pressure, maintain normal glucose tolerance and improve lipid balance [2], thereby reducing the risk of developing CHD [3]. Although PA is cardioprotective, different patterns of PA have different benefits for cardiovascular disease (CVD) prevention [4–6]. While CHD was responsible for more than 9 million deaths worldwide in 2019, and though CVD mortality has decreased in recent decades in Western countries, especially due to improved diagnostic and preventive methods, CHD continues to increase in developing countries, due to globalization and the adoption of more Western lifestyles [7, 8]. In Lebanon, CHD accounts for nearly 47% of total annual deaths [9].

Lack of PA has been identified as the fourth leading risk factor (RF) for global mortality and one of the leading public health indicators [10]. Physical inactivity is an important RF for CHD [11] and is the highest in high-income countries. Nowadays, high levels of physical inactivity are also observed in some middle-income countries, particularly among women [12].

It is known that physically active women seem less likely to develop CHD compared to inactive women [13–15]. However, in most countries, women are less active than men [16] and a third of women do not engage in leisure-time PA [3]. PA levels appear to decrease progressively after 65, with a greater decline among women [17]. Otherwise, sedentary time was associated in a dose–response relationship with an increased cardiovascular risk. On average, each additional hour of sedentary time in older women was associated with a 26% increase in adjusted CHD risk [18]. Replacing sitting with moderate or vigorous PA may be associated with reduced CVD mortality risk [19].

While women have CHD that is sometimes difficult to identify quickly (i.e., myocardial infarction/ischemia syndromes with no obstructive coronary artery disease (MINOCA/INOCA)) [20, 21], which may lead to a delay in their management and an unfavorable impact on their prognosis compared with men, few studies have focused on specifying the intensity and profile of PA that could be useful in preventing CHD in women.

According to the World Health Organization (WHO), currently 42% of Lebanese women are physically inactive [22], thus constituting a major public health problem [23].

The aim of our study is to identify the PA and sedentary pattern of Lebanese women in order to determine a potentially useful profile for the prevention of CHD in women.

## Methods

### Study design

The inclusions were performed from December 2018 to December 2019 in hospitals based in Beirut and Mount-Lebanon regions (Military Hospital, Makassed Hospital, Sacred Heart Hospital, Lebanese Geitaoui Hospital, Rafik Hariri University Hospital, and Mount-Lebanon Hospital).

We included, as previously described [24], 300 women aged 40 years and older, with a primary diagnosis of CHD in the cardiology service, following a myocardial infarction (MI) with or without ST-segment elevation or stable/unstable angina; diagnoses confirmed by a cardiologist based on their clinical presentation and paraclinical data. The case group had no history of heart disease (MI, CHD, valvular heart disease, cardiomyopathy, and myocarditis). For each case, four controls aged ≥ 40, matched by hospital, were randomly selected from surgical and general medicine wards, constituting a control group of 1200 patients (excluded were patients with a history of CHD, pregnant patients, or those suffering from cancer or mental disorders). Informed consent was obtained from each patient included, after validation of the protocol by the ethics committee of each hospital.

Calculation of total enrollment (*n* = 1500) was done by estimating the minimum sample size that would be necessary to show a twofold increase in CHD odds, in a case–control ratio of 1/4, based on CHD prevalence in Lebanese women older than 40 years (9%) [25], an alpha error of 5%, and a study power of 80% (Epi Info™).

### Data collection

After analysis of the medical records, allowing the collection of certain sociodemographic (age, weight, height) and medical (medical treatments, biological and paraclinical data) information, a face-to-face interview by the investigator was carried out.

We collected socio-demographic elements (residence area, educational level (low: illiterate or primary school; middle: complementary or secondary school; high: university level), marital status, professional status, monthly income), factors related to patients’ health, particularly cardiovascular: age, menopausal status, smoking (assessed based on tobacco, cigarette or waterpipe consumption (current smoker (smoked in the last 12 months), non-smoker and former smoker (quit smoking more than a year earlier))) [1, 26], RFs for CHD based on patient self-reported, current use of medications and/or laboratory test results when available: (hypertension (≥ 140/90 mmHg), dyslipidemia (non-HDLc (non-High-Density Lipoprotein Cholesterol) ≥ 3.4 mmol/L, triglycerides ≥ 1.7 mmol/L or LDLc (Low-Density Lipoprotein Cholesterol) ≥ 3 mmol/L [27], diabetes (random blood sugar ≥ 11.1 mmol/L or glycated hemoglobin ≥ 6.5% [28]), body mass index ((BMI), overweight: BMI of 25 to 29.9 kg/m2, obesity: BMI ≥ 30 kg/m^2^) [12], lifestyle factors (alcohol consumption (consumption of any alcoholic beverage within the previous 12 months) [29], diet). Eating habits were assessed using the Lebanese Mediterranean Diet Score (LMDS) (scores from 0 to 80 (best nutritional quality score)) [30]. Depression was assessed using the Beirut Distress Scale (BDS-22) (score from 0 to 66 (maximum psychological distress)) [31].

### Physical activity

PA was assessed using the International Physical Activity Questionnaire (IPAQ) long form version [32].

Total PA was calculated by giving each type of activity by its estimated energy requirements: 3.3 metabolic equivalent task (MET) for walking; 4 for moderate-intensity and 8 for vigorous PA (during work or leisure); 6 for cycling (transport); 4 and 5.5 for moderate- and heavy-intensity garden work, respectively; and 3 for moderate-intensity housework. Thus, the total PA score was obtained by multiplying the MET score by the minutes performed in a week for all types of activities in all domains (work, transportation, housework/gardening, leisure). A woman was considered moderately active for a score of 600 to 3000 MET-min/week combining all types of activities (equivalent to 5 or more days of moderate-intensity activity and/or walking of at least 30 min per day; or 20 min of vigorous activity 3 days per week, or a combination of both), and physically very active if the score was ≥ 3000 MET-min/week. A woman was classified as low active or inactive if she did not meet any of these criteria.

Sedentary time was further determined as the time reported to be at rest, other than sleep (such as sitting during transportation, work, or leisure, watching television, using a computer or cell phone) [33].

### Data analysis

Data were analyzed using SPSS version 21. Categorical variables are expressed in frequency and percentages, continuous variables in mean and standard deviation. Pearson’s chi-square test assessed the association between the different independent variables and the dependent variable (CHD status). Means were compared using the Independent Samples T-test. Patients were classified according to a minimum level of PA, with at least 600 MET-min/week defining a "physically active" status. Odds ratios (OR) and 95% confidence intervals (CI) were calculated. Multivariate logistic regression, using the enter method, assessed the association of PA domains and sedentary behavior with CHD, adjusted for covariates. To reduce the potential for multicollinearity, the domains of PA and sedentary time were entered as independent variables in the first block of the analysis and the other variables in the second block. Each variable having a *p*-value < 0.2 in the bivariate analysis was included in the models. The final model was accepted after checking the adequacy of the data using the Hosmer–Lemeshow test.

## Results

### Socio-demographic characteristics by CHD status

The baseline characteristics of the study population are presented in Table 1. Compared with controls, CHD women were significantly older (65.46 ± 10.33 years vs 62.36 ± 12.44 years, *p* < 0.001), lived more in the capital Beirut, in urban areas (28% vs 22.3%, *p* < 0.01), were unmarried (50.3% vs 43.8%, *p* < 0.05) and had a lower educational level (47.3% vs 36.8%, *p* < 0.01).

**Table 1:** Baseline socio-demographic characteristics of study population

**Characteristics**	**Total**	**Controls n (%)**	**Cases n (%)**	***p*-value**
	**1500 (100%)**	**1200 (80%)**	**300 (20%)**	
**Age, years (mean ± SD)**	62.98 ± 12.11	62.36 ± 12.44	65.46 ± 10.33	< 0.001***
**Governorate**				0.005**
Beirut	351 (23.4%)	267 (22.3%)	84 (28%)	
Mount Lebanon	693 (46.2%)	579 (48.3%)	114 (38%)	
Others^a^	456 (30.4%)	354 (29.5%)	102 (34%)	
**Marital status**				0.040*
Married	824 (54.9%)	675 (56.3%)	149 (49.7%)	
Divorced / widow / single	676 (45.1%)	525 (43.8%)	151 (50.3%)	
**Work status**				0.087
Yes	144 (9.6%)	123 (10.3%)	21 (7%)	
No/Retired	1356 (90.4%)	1077 (89.8%)	279 (93%)	
**Educational level**^**b**^				0.002**
Low	583 (38.9%)	441 (36.8%)	142 (47.3%)	
Middle	744 (49.6%)	610 (50.8%)	134 (44.7%)	
High	173 (11.5%)	149 (12.4%)	24 (8%)	
**Income level per individual**^**c**^				0.283
Lower class	64 (4.3%)	47 (3.9%)	17 (5.7%)	
Middle class	1054 (70.3%)	852 (71.0%)	202 (67.3%)	
Higher class	382 (25.5%)	301 (25.1%)	81 (27%)	

Continuous variables are presented as mean ± standard deviation (SD) and categorical variables are presented as frequencies (percentages)

The Independent Samples T-test was used to compare continuous variables, while Pearson’s chi-square test to compare categorical variables

^*^p-value ≤ 0.05

^**^p-value ≤ 0.01

^***^p-value ≤ 0.001

^a^Others regions of Lebanon: South Lebanon, North/Akkar, Bekaa, Baalback/Hermel and Nabatieh

^b^Educational level: low, illiterate or primary school; middle, complementary or secondary school; high, university level

^c^Monthly income per individual: low, < 180 LBP/month/person; middle, 180–675 LBP/month/person; high, > 675 LBP/month/person (LBP: Lebanese pound)

### Health characteristics by CHD status

Table 2 shows the health-related characteristics of the study population. The postmenopausal status seemed to be associated with CHD in women (94% vs 85.5% in coronary cases and controls, respectively, *p* < 0.001). Patients with CHD had also more cardiovascular RFs compared to controls (hypertension (91.3% vs 68.4%, respectively, *p* < 0.001), dyslipidemia (78% vs 48.7%, respectively, *p* < 0.001) and diabetes (54% vs 36.5%, respectively, *p* < 0.001)).

**Table 2:** Health related characteristics of study participants

**Characteristics**	**Total**	**Controls n (%)**	**Cases n (%)**	***p*-value**
	**1500 (100%)**	**1200 (80%)**	**300 (20%)**	
**Postmenopausal status**				< 0.001**
No	192 (12.8%)	174 (14.5%)	18 (6.0%)	
Yes	1308 (87.2%)	1026 (85.5%)	282 (94.0%)	
**BMI**				0.385
Underweight / Normal	369 (24.6%)	300 (25.0%)	69 (23.0%)	
Overweight	484 (32.3%)	393 (32.8%)	91 (30.3%)	
Obese	647 (43.1%)	507 (42.3%)	140 (46.7%)	
**Hypertension**				< 0.001**
No	405 (27.0%)	379 (31.6%)	26 (8.7%)	
Yes	1095 (73%)	821 (68.4%)	274 (91.3%)	
**Dyslipidemia**				< 0.001**
No	682 (45.5%)	616 (51.3%)	66 (22.0%)	
Yes	818 (54.5%)	584 (48.7%)	234 (78.0%)	
**Diabetes**				< 0.001**
No	900 (60.0%)	762 (63.5%)	138 (46.0%)	
Yes	600 (40.0%)	438 (36.5%)	162 (54.0%)	
**Smoking status**^**a**^				< 0.001**
Never / past	837 (55.8%)	701 (58.4%)	136 (45.3%)	
Current	663 (44.2%)	499 (41.6%)	164 (54.7%)	
**Alcohol consumption**				0.586
No	1367 (91.1%)	1096 (91.3%)	271 (90.3%)	
Yes	133 (8.9%)	104 (8.7%)	29 (9.7%)	
**LMDS, mean** ± **SD**	40.15 ± 5.20	40.30 ± 5.08	39.53 ± 5.64	0.031*
**BDS-22, mean** ± **SD**	11.27 ± 14.13	10.88 ± 13.65	12.85 ± 15.86	0.048*
**Rheumatic diseases**				0.540
No	1430 (95.3%)	1142 (95.2%)	288 (96.0%)	
Yes	70 (4.7%)	58 (4.8%)	12 (4.0%)	
**Declared joint pain**				< 0.001**
No	615 (41.0%)	525 (43.8%)	90 (30.0%)	
Yes	885 (59.0%)	675 (56.3%)	210 (70.0%)	

Continuous variables are presented as mean ± standard deviation (SD) and categorical variables are presented as frequencies (percentages)

The Independent Samples T-test was used to compare continuous variables, while Pearson’s chi-square test to compare categorical variables

*BMI* body mass index, *LMDS* Lebanese Mediterranean Diet Score, *BDS* Beirut Distress Scale

^*^p-value ≤ 0.05

^**^p-value ≤ 0.001

^a^Smoking status corresponds to cigarettes and/ or waterpipes consumption: current smoker (smoked in the last 12 months), non-smoker and former smoker (quit smoking more than a year earlier)

Regarding lifestyle habits, smoking, which involves more than 44% of the study patients, was significantly higher among coronary cases (54.7% vs 41.6%, respectively, *p* < 0.001); in addition to the non-adherence to a healthy Mediterranean diet (39.53 ± 5.64 vs 40.30 ± 5.08, respectively, *p* < 0.05) and psychological distress (12.85 ± 15.86 vs 10.88 ± 13.65, respectively, *p* < 0.05).

It is interesting to note that cases also suffered significantly more from common joint pain (very frequent in our sample (59%) and likely to discourage PA) (70% vs 56.3%, respectively, *p* < 0.001).

### PA and coronary odds

We studied the PA pattern (intensity and domains) in our study’s patients (Table 3).

**Table 3:** Physical activity rates and coronary heart disease odds according to different types and domains of physical activity

	**Controls n (%)**	**Cases n (%)**	**Chi-square *p*-value**	**OR (95%CI)**	***p*-value**
***Frequency***	1200 (80%)	300 (20%)			
***Total PA***^***a***^***, n (%)***			< 0.001***		
Physically inactive^b^	476 (39.7%)	160 (53.3%)		1.00 (Ref.)	
Physically active^c^	724 (60.3%)	140 (46.7%)		0.575 (0.446–0.742)	< 0.001***
***Types of PA, n (%)***					
***Total walking***^*d*^			0.453		
< 60 min/week	730 (60.8%)	193 (64.3%)		1.00 (Ref.)	
60–180 min/week	157 (13.1%)	39 (13.0%)		0.940 (0.639–1.381)	0.751
> 180 min/week	313 (26.1%)	68 (22.7%)		0.822 (0.605–1.116)	0.209
***Total PA of moderate intensity***^***c***^			< 0.001***		
< 60 min/week	408 (34.0%)	151 (50.3%)		1.00 (Ref.)	
60–180 min/week	156 (13.0%)	38 (12.7%)		0.658 (0.441–0.983)	0.041*
> 180 min/week	636 (53.0%)	111 (37.0%)		0.472 (0.358–0.621)	< 0.001***
***Total PA of vigorous intensity***^***f***^			0.784		
< 60 min/week	1179 (98.3%)	297 (99.0%)		1.00 (Ref.)	
60–180 min/week	10 (0.8%)	2 (0.7%)		0.794 (0.173–3.643)	0.767
> 180 min/week	11 (0.9%)	1 (0.3%)		0.361 (0.046–2.806)	0.330
***Domains of PA, n (%)***					
***Work Domain***			0.384		
Unemployed and low amount	1151 (95.9%)	291 (97.0%)		1.00 (Ref.)	
Moderate and high amount	49 (4.1%)	9 (3.0%)		0.726 (0.353–1.496)	0.386
***Transportation Domain***			< 0.001***		
Low amount	1002 (83.5%)	281 (93.7%)		1.00 (Ref.)	
Moderate amount	183 (15.3%)	18 (6.0%)		0.351 (0.212–0.579)	< 0.001***
High amount	15 (1.3%)	1 (0.3%)		0.238 (0.031–1.807)	0.165
***Domestic and Garden Domain***			< 0.001***		
Low amount	583 (48.6%)	204 (68.0%)		1.00 (Ref.)	
Moderate amount	544 (45.3%)	92 (30.7%)		0.483 (0.368–0.635)	< 0.001***
High amount	73 (6.1%)	4 (1.3%)		0.157 (0.057–0.434)	< 0.001***
***Leisure-time Domain***			0.081		
Low amount	1074 (89.5%)	281 (93.7%)		1.00 (Ref.)	
Moderate amount	114 (9.5%)	18 (6.0%)		0.603 (0.361–1.009)	0.054
High amount	12 (1.0%)	1 (0.3%)		0.319 (0.041–2.460)	0.273

Low amount: < 600 MET-minutes/week; moderate amount: 600–3000 MET-minutes/week; high amount: ≥ 3000 MET-minutes/week

Pearson’s chi-square test was used to compare the differences between cases and controls, and binary logistic regressions to calculate the OR, 95% CI and the corresponding *p*-value

*PA* physical activity, *OR* odds ratio, *CI* confidence interval, *MET* metabolic equivalent task

^*^p-value ≤ 0.05

^**^p-value ≤ 0.01

^***^p-value ≤ 0.001

^a^Total PA: sum of work, transportation, domestic and garden and leisure scores of IPAQ (International Physical Activity Questionnaire), expressed in MET-minutes/week

^b^Physically inactive: none or low-intensity (< 600 MET-min/week)

^c^Physically active: at least moderate-intensity (≥ 600 MET-min/week)

^d^Total walking: sum of weekly walking activities at work, for transportation and in leisure-time, in minutes/week

^e^Total PA of moderate intensity: sum of weekly moderate activities at work, in domestic and garden chores, in leisure-time and by cycling for transportation, and vigorous garden chores, in minutes/week

^f^Total PA of vigorous intensity: sum of weekly vigorous activities at work and in leisure-time, in minutes/week

53.3% of hospitalized patients with newly diagnosed CHD reported no or low PA before hospitalization, whereas 60.3% of patients in the control group engaged in at least moderate activity (OR = 0.575 [0.446–0.742], *p* < 0.001). While walking or vigorous PA did not appear to be significantly different between CHD patients and controls, moderate PA appeared to be associated with significantly reduced odds of developing CHD with a graded response to weekly practice duration. Half of the coronary cases (50.3%) were involved in less than 60 min/week of moderate-intensity activity, while 53% of the controls were engaging in more than 180 min/week of such activity. Women practicing moderate activity for 60–180 min/week or more than 180 min/week were less likely to be at risk than those who practiced less than 1 h/week (OR = 0.658 [0.441–0.983], *p* < 0.05, and OR = 0.472 [0.358–0.621], *p* < 0.001, respectively).

Concerning the PA domains, activities inherent to the work do not seem to be very different. Similarly, leisure-time PA did not appear to differ significantly between the two patient groups. On the other hand, PA for transportation and domestic or gardening work seemed significantly different between the 2 groups. Over 15% of non-coronary women were engaged in moderate transportation-related PA (600–3000 MET-min/week), compared with 6% of coronary women, representing a 64.9% reduction in the associated CHD threat (OR = 0.351 [0.212–0.579], *p* < 0.001). In addition, a 'dose–response' relationship in household or gardening activities was also associated with a reduced odds of CHD in active women: 30.7% of cases vs 45.3% of controls practiced this type of activity at moderate amount (600–3000 MET-min/week), and 1.3% of cases vs 6.1% of controls at high amount (≥ 3000 MET-min/week), thus protecting 51.7% and 84.3% against CHD (OR = 0.483 [0.368–0.635]; and 0.157 [0.057–0.434], *p* < 0.001, respectively).

### Sedentary lifestyle and PA

**Fig. 1:**
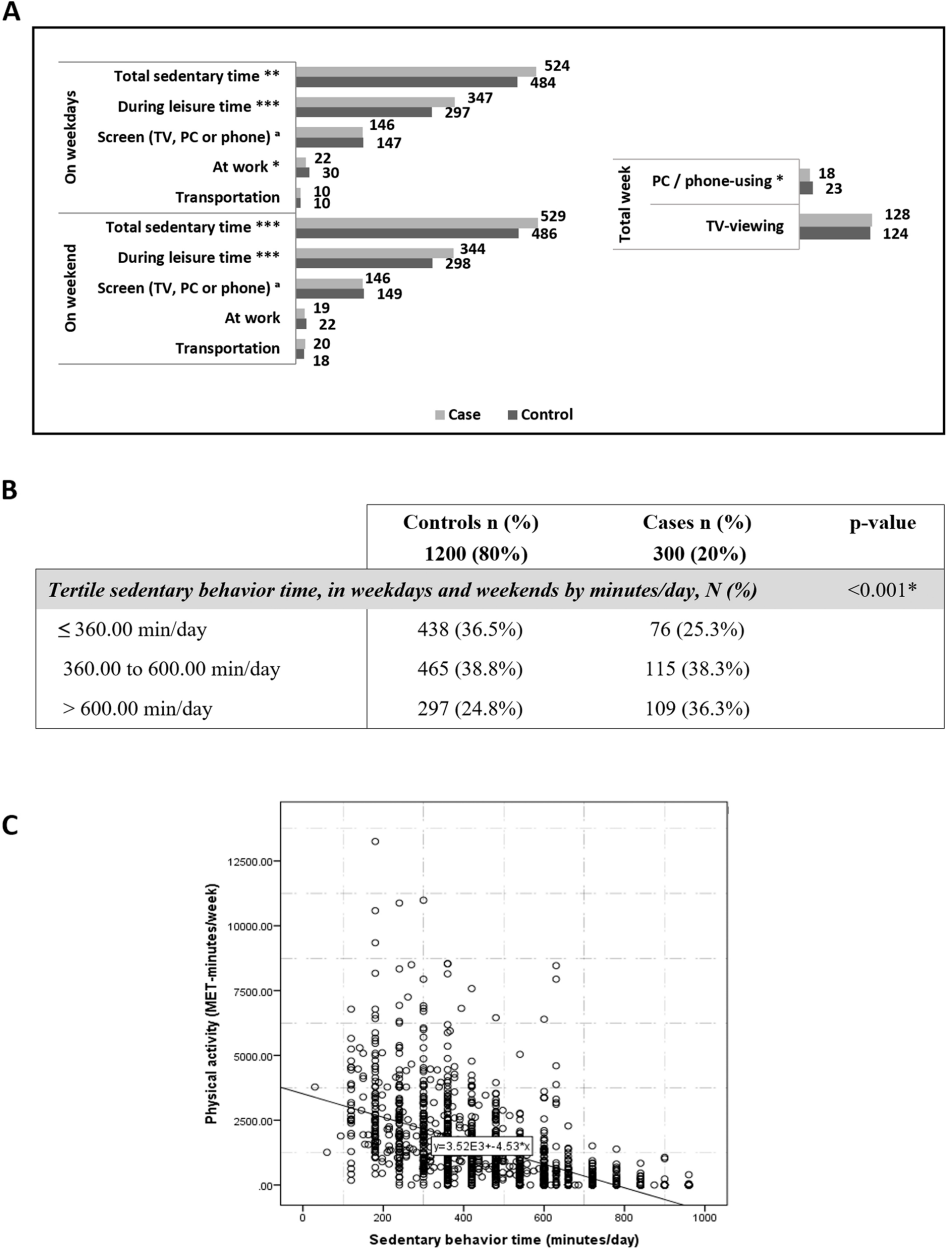
Sedentary in study participant. **A** Sedentary time and domains of sedentary in study participants. Average time (minutes/day) reportedly spent sitting in each domain on a usual weekday and weekend day (left) and in front of a screen over the week (right) among coronary and control women. ^a^Screen represents the sum of TV-viewing and PC or phone-using. **B** Comparison between controls and coronary cases for the total sedentary behavior time (minutes/day) during the week. **C** Scatter plot for the relationship between sitting time (minutes/day) and physical activity (MET-minutes/week). Pearson’s correlation coefficient = -0.580 (*P* < 0.001). *p-value ≤ 0.05, **p-value ≤ 0.01, ***p-value ≤ 0.001

We assessed the sedentary profile of our study’s patients (Fig. 1A). The total daily sedentary time (in minutes/day) emerged significantly different between the two groups, on weekdays and weekends (524 ± 206 vs 484 ± 204, *p* < 0.01 and 529 ± 205 vs 486 ± 202, *p* = 0.001, respectively); essentially corresponded to substantially more leisure time spent sitting on weekdays (347 ± 219 vs 297 ± 200, *p* < 0.001) and weekends (344 ± 219 vs 298 ± 198, *p* = 0.001) in coronary patients compared with controls (Fig. 1A, left). In contrast, controls tended to spend more time sitting at work during the week (30 ± 75 vs 22.52 ± 60, *p* = 0.049).

Note that the domain usually taken as a reference to assess sedentary time (screen time) did not appear significant between coronary women and controls if total screen time was measured (Fig. 1A, right). A total of 36.3% of coronary women spent more than 10 h a day sitting during the week (weekdays and weekends), compared with 24.8% of non-coronary women (Fig. 1B), which could increase their odds of developing CHD.

An inverse significant correlation was found between PA level and reported sedentary time in the women studied (Pearson coefficient = -0.580, *p* < 0.001) (Fig. 1C).

### PA, sedentary lifestyle and coronary odds

**Fig. 2:**
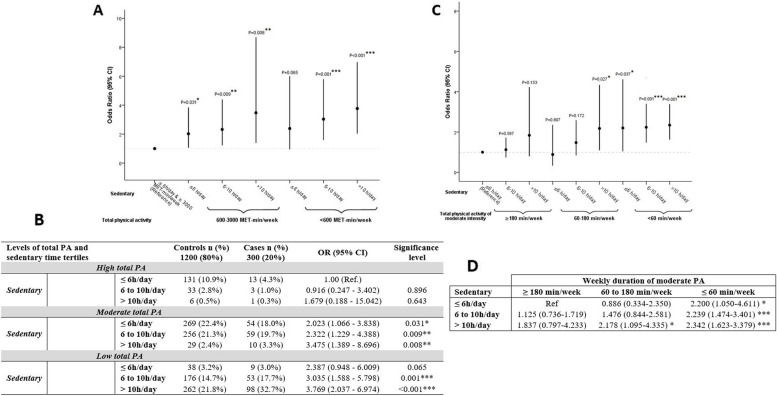
Odds ratio of daily sedentary associated with weekly PA and the odds of CHD. **A**, **B** Sedentary and total PA; *C, D*. Sedentary and duration of total PA of moderate intensity. **A**, **C** Schematic representation of ORs with 95%CI. **B**, D Table including the numerical values of the ORs (95%CI). The reference category is the best condition group: lowest levels of sedentary (≤ 6 h/day) and higher weekly total PA (≥ 3000 MET-min/week) (**A**, **B**), lowest levels of sedentary (≤ 6 h/day) and higher weekly duration of moderate-intensity PA (≥ 180 min/week) (**C**, **D**). PA: physical activity, OR: Odds ratio, CI: Confidence interval, MET: metabolic equivalent task, CHD: coronary heart disease. *p-value ≤ 0.05, **p-value ≤ 0.01, ***p-value ≤ 0.001

The summary ORs for the joint associations of sitting time and total PA with CHD odds are shown in Fig. 2. A clear dose–response association was observed, with an increased odds of CHD with increasing sitting time in combination with lower activity levels (Fig. 2A and B). Compared with those engaging in high total PA and having the least sedentary lifestyle (sitting ≤ 6 h/day), patients having moderate PA and low PA levels were more likely to be in the CHD group, with increasing odds as the sedentary time increases (Fig. 2B), suggestive of the impact of prolonged sitting on PA on CHD development. Of note, very few women in our study practiced weekly PA of ≥ 3000 MET-min/week.

Also, we specifically assessed the association between weekly moderate-intensity PA duration, sedentary time, and coronary odds (Fig. 2C and D): weekly moderate-intensity PA of 1 to 3 h was accompanied by an increased odds of CHD with a daily sedentary time greater than 10 h (Fig. 2C) (OR = 2.178 [1.095–4.335], *p* < 0.05) (Fig. 2D). Below 1 h of weekly moderate PA, the odds of CHD was significantly increased regardless of the sedentary time level (Fig. 2C and D), but more so the higher the daily sedentary time (OR = 2.200 [1.050–4.611], *p* < 0.05; OR = 2.239 [1.474–3.401], *p* = 0.001; OR = 2.342 [1.623–3.379], *p* = 0.001, respectively for increasing duration of sedentary time) compared with a moderate PA level of at least 3 h in a woman spending less than 6 h per day sitting. (Fig. 2C and D).

**Fig. 3:**
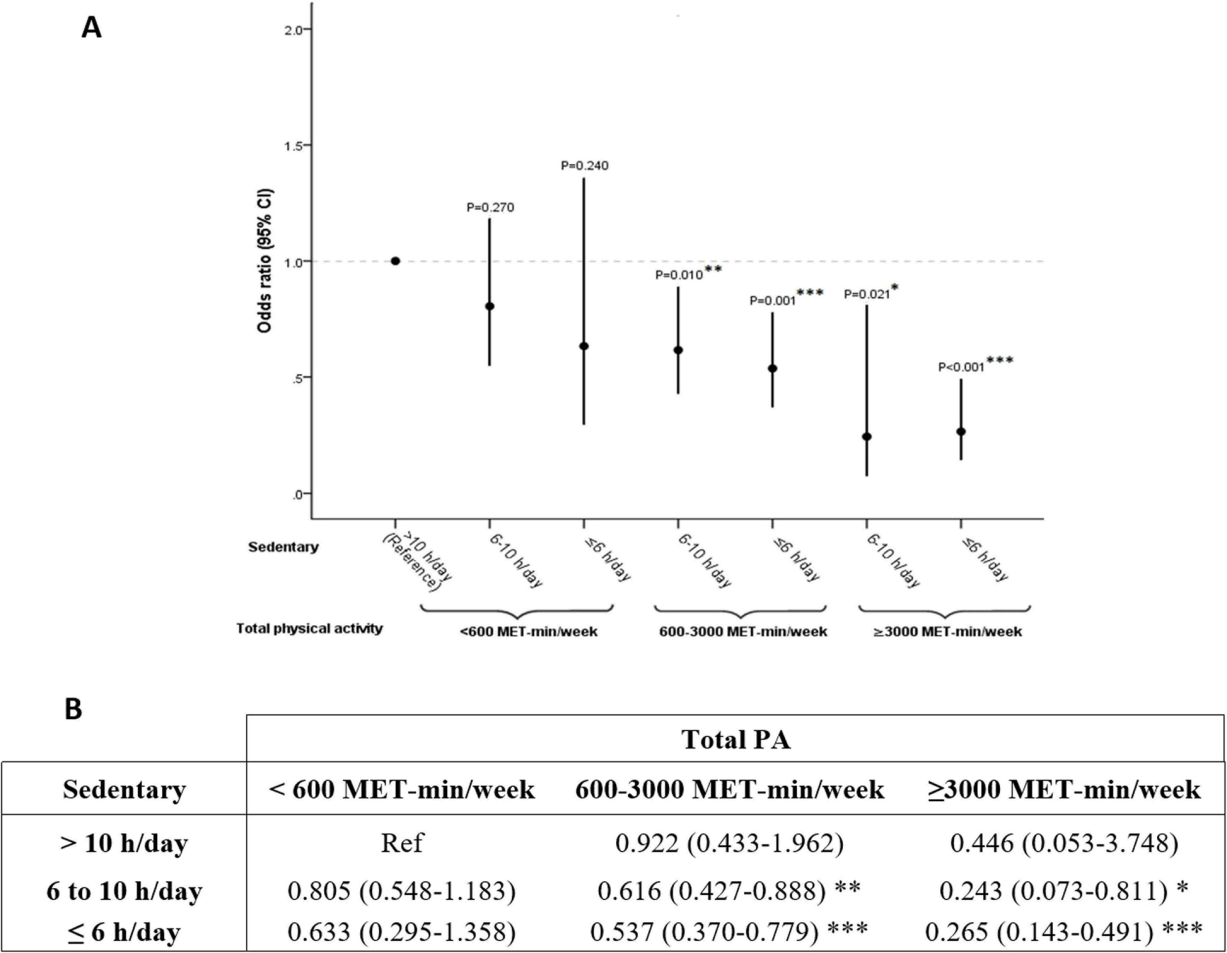
Odds ratio of daily sedentary associated with weekly total PA level and the odds of CHD. **A** Schematic representation of ORs with 95%CI. **B** Table including the numerical values of the ORs (95%CI). The reference category is the group with the highest levels of sitting time (> 10 h/day) and lowest total PA (< 600 MET-min/week). PA: physical activity, OR: odds ratio, CI: confidence interval, MET: metabolic equivalent task, CHD: coronary heart disease. *p-value ≤ 0.05, **p-value ≤ 0.01, ***p-value ≤ 0.001

In contrast to these results, the detrimental influence of sedentary lifestyle on coronary odds appeared to be reversed by weekly PA of moderate frequency and duration (600–3000 MET-min/week), especially as sedentary lifestyle was less (OR = 0.616 [0.427–0.888], *p* = 0.01; OR = 0.537 [0.37–0.779], *p* = 0.001, respectively for sedentary time of 6 to 10 h and ≤ 6 h weekly) and as soon as sedentary time did not exceed 10 h daily (OR = 0.922 [0.433–1.962], ns) (Fig. 3A and B).

The odds of CHD (adjusted OR and 95%CI) in women associated with PA domains and sedentary time was assessed in different models: after adjustment for sociodemographic factors (model 1), further combining adjustment for joint pain, and depression (model 2), and finally adjusted for smoking, LMDS, and biological RFs (model 3) (Table 4).

**Table 4:** Association between domains related physical activity, sedentary time and CHD among women

**PA and Sedentary time**	**Model 1**^**a**^	***P*-value**	**Model 2**^**b**^	***P*-value**	**Model 3**^**c**^	***P*-value**
	**Adjusted OR (95% CI)**		**Adjusted OR (95% CI)**		**Adjusted OR (95% CI)**	
***Transportation Domain***						
Low amount^d^	_	0.001***	_	0.007**	_	0.006**
Moderate amount^e^	0.392 (0.236–0.653)	< 0.001***	0.449 (0.267–0.755)	0.003**	0.426 (0.247–0.734)	0.002**
High amount^f^	0.266 (0.034–2.060)	0.205	0.343 (0.044–2.688)	0.308	0.346 (0.041–2.930)	0.331
***Domestic and Garden Domain***						
Low amount^d^	_	< 0.001***	_	< 0.001***	_	0.001***
Moderate amount^e^	0.528 (0.395–0.707)	< 0.001***	0.562 (0.400–0.790)	0.001***	0.566 (0.396–0.808)	0.002**
High amount^f^	0.164 (0.058–0.461)	0.001***	0.162 (0.056–0.473)	0.001***	0.193 (0.065–0.578)	0.003**
***Leisure-time Domain***						
Low amount^d^	_	0.191	_	0.532	_	0.680
Moderate amount^e^	0.638 (0.380–1.073)	0.090	0.756 (0.444–1.287)	0.303	0.823 (0.468–1.448)	0.500
High amount^f^	0.476 (0.060–3.767)	0.482	0.603 (0.076–4.810)	0.633	0.528 (0.061–4.540)	0.561
***Sedentary time, h/day)***						
≤ 6 h/day	_	0.007**	_	0.038*	_	0.089
6 to 10 h/day	1.353 (0.975–1.875)	0.070	1.325 (0.953–1.841)	0.094	1.212 (0.858–1.712)	0.275
> 10 h/day	1.778 (1.243–2.542)	0.002**	1.609 (1.116–2.318)	0.011*	1.533 (1.046–2.247)	0.029*

The dependent variable was coronary heart disease status (cases vs controls (ref))

*PA* physical activity, *OR* odds ratio, *CI* confidence interval, *MET* metabolic equivalent task

^*^p-value ≤ 0.05

^**^p-value ≤ 0.01

^***^p-value ≤ 0.001

^a^ Regression model 1 adjusted for sociodemographic factors: age, governorate, education, work and marital status

^b^ Regression model 2 adjusted for variables included in model 1, plus joint pain and depression (BDS-22 score)

^c^ Regression model 3 adjusted for variables included in model 2, plus smoking, Lebanese Mediterranean Diet Score, and biological (menopause, dyslipidemia, hypertension, diabetes) risk factors

^d^Low amount: < 600 MET-minutes/week; ^e^moderate amount: 600 to 3000 MET-minutes/week; ^f^high amount: ≥ 3000 MET-minutes/week

Leisure-time PA and vigorous transportation-related PA were associated with decreased but non-significant odds of CHD. In contrast, moderate transportation-related PA was correlated with a significant decrease in the odds of CHD in model 1, and remained statistically significant after adjustment for sociodemographic, lifestyle, and cardiovascular RFs (model 3) (OR = 0.426 [0.247–0.734], *p* = 0.002, compared with low PA in the context of transportation.

PA related to housework or gardening was associated with a significantly lower odds of CHD, with a substantial reduction of 43.4% and 80.7% observed for moderate- (600–3000 MET-min/week) and high-PA (≥ 3000 MET-min/week), respectively, compared with those with little or no exercise. In contrast, women who sat for long periods of time (> 10 h/day) had a significant increase of approximately 53.3% in the odds of CHD (OR = 1.533 [1.046–2.247], *p* = 0.029) compared with those sitting less than 6 h/day.

## Discussion

To our knowledge, our study is the first to assess the relationship between PA and odds of CHD in Lebanese women. It is also the first to provide a unique insight into the different patterns of PA and sedentary behavior in Lebanese women with CHD. Essentially, our results showed that women who practice at least moderate-intensity PA, both in transportation and in conventional activities of daily living, appeared to be protected against CHD. On the other hand, a significant sedentary lifestyle in women, more than 10 h of daily sitting, was associated with an increased odd of CHD.

The rapid sociodemographic transition in developing countries has introduced substantial lifestyle changes that have been largely characterized by increasing prevalence of obesity and physical inactivity [34] and consequently increasing risk of CVD [8]. In general, low or declining PA levels often correspond to high or rising gross national product [3, 16].

The prevalence of physical inactivity among women in our study (42.4%) was very close to that of non-communicable diseases (41.7%) published in 2010 by WHO. This result was consistent with other studies showing that most women in Arab countries suffer from insufficient PA, for example, 76.2% in Saudi Arabia, 72.1% in Kuwait, 68.9% in the Emirates, 47.6% in Mauritania, 54% in Iraq, and 40.3% in Tunisia [12, 35].

We did not find a significant protective association of leisure-time PA or high-amount transport PA with CHD, which is puzzling compared with the literature [36, 37]. This could be explained by the low prevalence of high-amount PA for transport in our population, possibly due to the predominance of hot, sunny weather limiting this type of activity. In addition, there is a barrier related to the lifestyle habits, most of the women live in urban/peri-urban areas (Beirut and Mount-Lebanon), thus with a lack of green spaces for regular PA practice, and Lebanese women are generally not accustomed to using gyms, as demonstrated in Polish women who do not practice enough during their leisure time [38]. However, moderate PA in the transport setting resulted in a substantial reduction in the odds of coronary events among women studied, a result consistent with previous studies [13, 15] showing that intense activities were not necessary to reduce the rate of CHD in women.

Work-related PA did not appear to be associated with CHD in our study, in line with the results of a previous study [39], which is quite old, but perhaps consistent with the low proportion of working women in our study (144 patients (9.6% of the total number), of whom only 21 were CHD patients (1.4%)), and on the other hand infrequently in physically demanding jobs. However, it should be noted that previous research had not suggested a greater potential protective benefit for vigorous-intensity PA [4], moreover, intense occupational PA could be detrimental to health [40, 41].

Previous study suggested that sitting for 10 or more hours per day was associated with increased odds of CVD [42] and mortality, but at least moderate PA could reverse this adverse effect [19]. However, sedentary behavior is not simply the absence of PA. Individuals may engage in PA while otherwise spending a lot of time sitting [43]. In our study, women with CHD had significantly more sedentary time than controls, including after adjustment for confounders. We note the protective role of various household and/or gardening activities on CHD. These results are interesting, because domestic activity remains the main contributor to daily PA, especially in the elderly [44]. These findings are consistent with previous studies showing that home-based activity has health benefits, by reducing cardiovascular mortality [5, 45] and CHD risk [5]. Gardening can, moreover, be discussed as an integral part of the "Mediterranean diet" combining an omega-3 and vitamin rich, protective dietary culturally adopted in Mediterranean countries (which our patients present), and outdoor moderate daily PA, favoring vitamin D synthesis, seasonal adaptation to light, consumption of mature fruits and vegetables; this combined strategy improves women’s health [46].

One study noted that PA related to housework was not associated with a reduced odd of CVD [47], but the mean age was lower (52.4 years) and the assessment was for intense domestic PA only. Although retired or unemployed women were less likely to participate in PAs during their leisure time, they appeared to spend more time on housework, as shown in Brownson’s study [48].

Thus, our results in Lebanese women are consistent with recent European Society of Cardiology (ESC) guidelines recommending adults of all ages to strive for at least 150–300 min a week of moderate-intensity PA [49], and could be used in a pragmatic CVD prevention strategy in aging Lebanese women, as has been considered for other countries [50].

Our study has some limitations. The sample was composed of hospitalized patients from 2 regions and therefore may not represent a balanced distribution of the overall population. However, to minimize the selection bias effect, controls were selected from the same hospital as the cases. Self-reported PA likely has a significant measurement error [51], which may lead to underestimate the influence of PA on CHD odds. Although we performed a multivariate analysis, the possibility of residual confounding by unmeasured factors remains. However, the large sample size and face-to-face interviews increased the precision of the study. The use of incident cases also avoids survival bias. In addition, the assessment of all domains of daily PA enhances reflection on the outcomes. Furthermore, we collected detailed information on demographic, socioeconomic, and health factors, which allowed adjustment for these important confounders.

## Conclusion

Our results highlight the cardiovascular health benefits of PA in preventing CHD odds in Lebanese women, even while spending several hours sitting. As urbanization continues, promoting the potential benefits of easily accessible PA could be an important public health message for aging women who do not participate in PA in a sports club setting. Thus, actions to raise women’s awareness through the commitment of dedicated government policies could promote the virtuous couple: a Mediterranean diet associated with regular PA, accessible and adapted to the female population in developing countries for the benefit of their cardiovascular health.

## Data Availability

The datasets used and/or analyzed during the current study are available from the corresponding author on reasonable request.

## Abbreviations

BDS: Beirut Distress Scale
BMI: Body mass index
CHD: Coronary heart disease
CI: Confidence interval
CVD: Cardiovascular disease
ESC: European Society of Cardiology
INOCA: Ischemia with no obstructive coronary artery disease
IPAQ: International Physical Activity Questionnaire
LDLc: Low-Density Lipoprotein Cholesterol
LMDS: Lebanese Mediterranean Diet Score
MET: Metabolic equivalent
MI: Myocardial infarction
MINOCA: Myocardial infarction with no obstructive coronary artery disease
non-HDLc: Non-High-Density Lipoprotein Cholesterol
ns: Not significant
OR: Odds ratio
PA: Physical activity
RF: Risk factor
SPSS: Statistical Package for the Social Sciences
WHO: World health organization
